# Crystal Structures of the Plant Phospholipase A1 Proteins Reveal a Unique Dimerization Domain

**DOI:** 10.3390/molecules27072317

**Published:** 2022-04-02

**Authors:** Yunseok Heo, Inhwan Lee, Sunjin Moon, Ji-Hye Yun, Eun Yu Kim, Sam-Yong Park, Jae-Hyun Park, Woo Taek Kim, Weontae Lee

**Affiliations:** 1 Structural Biochemistry & Molecular Biophysics Laboratory, Department of Biochemistry, College of Life Science and Biotechnology, Yonsei University, Seoul 03722, Korea; uoonsek1@yonsei.ac.kr (Y.H.); inhwan@kolmar.co.kr (I.L.); moon@spin.yonsei.ac.kr (S.M.); jihye2@spin.yonsei.ac.kr (J.-H.Y.); jhpark@spin.yonsei.ac.kr (J.-H.P.); 2 PCG-Biotech, Ltd., 508 KBIZ DMC Tower, Sangam-ro, Seoul 03929, Korea; 3 Department of Systems Biology, College of Life Science and Biotechnology, Yonsei University, Seoul 03722, Korea; eunyukim@cemps.ac.cn; 4 Drug Design Laboratory, Graduate School of Medical Life Science, Yokohama City University, Tsurumi, Yokohama 230-0045, Japan; park@yokohama-cu.ac.jp

**Keywords:** X-ray crystallography, phospholipase A1, homodimer, dimerization domain, catalytic triad, plant protein

## Abstract

Phospholipase is an enzyme that hydrolyzes various phospholipid substrates at specific ester bonds and plays important roles such as membrane remodeling, as digestive enzymes, and the regulation of cellular mechanism. Phospholipase proteins are divided into following the four major groups according to the ester bonds they cleave off: phospholipase A1 (PLA1), phospholipase A2 (PLA2), phospholipase C (PLC), and phospholipase D (PLD). Among the four phospholipase groups, PLA1 has been less studied than the other phospholipases. Here, we report the first molecular structures of plant PLA1s: AtDSEL and CaPLA1 derived from *Arabidopsis thaliana* and *Capsicum annuum*, respectively. AtDSEL and CaPLA1 are novel PLA1s in that they form homodimers since PLAs are generally in the form of a monomer. The dimerization domain at the C-terminal of the AtDSEL and CaPLA1 makes hydrophobic interactions between each monomer, respectively. The C-terminal domain is also present in PLA1s of other plants, but not in PLAs of mammals and fungi. An activity assay of AtDSEL toward various lipid substrates demonstrates that AtDSEL is specialized for the cleavage of *sn*-1 acyl chains. This report reveals a new domain that exists only in plant PLA1s and suggests that the domain is essential for homodimerization.

## 1. Introduction

Phospholipase hydrolyzes phospholipids at specific sites. The phospholipases have roles as digestive enzymes, for maintaining and remodeling of a membrane, and for the regulation of cellular mechanisms [1]. The phospholipases are classified into four groups according to the cleavage site; phospholipase A1 (PLA1) cleaves *sn*-1 acyl chain, phospholipase A2 (PLA2) cleaves *sn*-2 acyl chain, phospholipase C (PLC) cleaves before the phosphate, and phospholipase D (PLD) cleaves after the phosphate [2]. Therefore, PLA1 and PLA2 are the acylhydrolase family that catalyzes the substrates containing acyl group, and PLC and PLD belong to phosphodiesterase. Triacylglycerol lipase (TGL), lipoprotein lipase (LPL), and monoacylglycerol lipase (MGL) belong to PLA because they also cleave the *sn*-1 and/or *sn*-2 acyl chain.

PLA1 (EC 3.1.1.32) is present in many different organisms including mammals, fungi, insects, bacteria, metazoans, protozoan parasites, venoms, and plants [1,3]. To date, three-dimensional structures of various PLAs have been elucidated [4,5,6,7,8,9,10]. Although these lipases have little sequence identity, they show a typical α/β hydrolase scaffold comprising of a core of seven (or more) β-strands flanked by several α-helices [11]. Other common structural features of lipases are a GXSXG motif, lid domain, and catalytic triad in the α/β hydrolase scaffold [12]. The GXSXG motif is composed of catalytic Ser and two residues before and after the Ser, and the lid domain plays a role for capping the active site; the catalytic triad consists of His, Asp, and the Ser of the GXSXG motif [13,14,15,16].

Plant PLAs have important roles in metabolism and lipid biosynthesis, as well as applications in food and biotechnology industries [17,18]. In *Arabidopsis thaliana*, DAD1 (At2g44810) was identified as an *sn*-1 specific acylhydrolase, and several studies have suggested that AtDSEL (*Arabidopsis thaliana* DAD1-like seedling establishment-related lipase), one of the homologues of DAD1, plays significant roles in the regulation of cellular processes such as seed germination, tissue growth, and seedling establishment [19,20]. In the early roots of hot pepper (*Capsicum annuum*), a cDNA encoding PLA1 homolog (CaPLA1) was identified [21]. CaPLA1 was selectively expressed in young roots of the hot pepper and hydrolyzed phospholipids at the *sn*-1 position [21]. CaPLA1 is presumed to be involved in the development of the roots of hot pepper, considering that the expression of CaPLA1 rapidly declined after germination [21].

We have determined the three-dimensional structures of AtDSEL and CaPLA1. They are the first elucidated structures of PLA1 derived from plants. Here, we revealed that these representative plant PLA1s are different from previously reported PLAs; only the plant PLA1s have a domain for homodimerization. Hydrophobic residues in the dimerization domain form a strong dimeric interface. This report broadens our knowledge of PLA by presenting the plant PLA1 structures with novel features.

## 2. Results and Discussion

### 2.1. Structural Features of AtDSEL and CaPLA1

**Figure 1:**
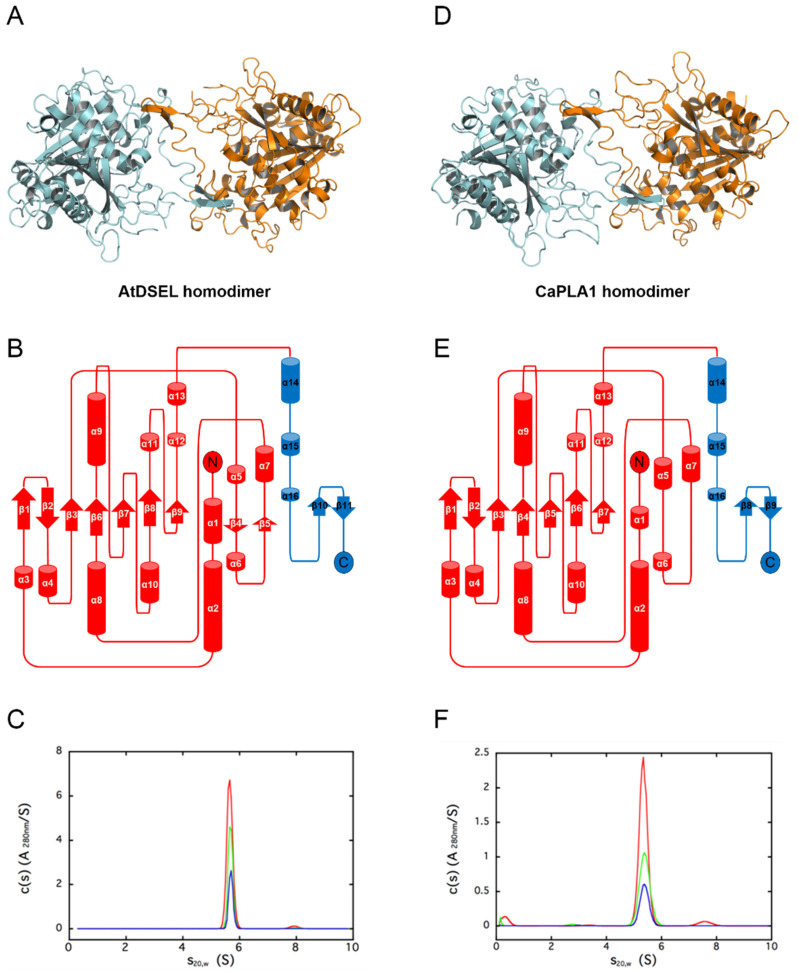
Overall structures of AtDSEL and CaPLA1. (**A**) Crystal structure of full-length AtDSEL. AtDSEL homodimer is shown as a ribbon diagram. Each monomer is colored in cyan and orange, respectively. (**B**) Topology diagram of AtDSEL monomer. α/β hydrolase domain and C-terminal domain are colored in red and blue, respectively. (**C**) Analytical ultracentrifugation (AUC) result of AtDSEL. The experiments were performed at following three protein concentrations: 3.0 mg/mL (colored in red), 1.5 mg/mL (green), and 0.8 mg/mL (blue). Calculated molecular weight of AtDSEL from the AUC peak is 95.3 ± 0.3 kDa, which strongly suggests that AtDSEL forms homodimers in solution, considering that the theoretical molecular weight of dimeric AtDSEL is 95.7 kDa. (**D**) Crystal structure of full-length CaPLA1. CaPLA1 homodimer is shown as a ribbon diagram. (**E**) Topology diagram of CaPLA1 monomer. (**F**) AUC result of CaPLA1. The experiments were performed at following three protein concentrations: 1.1 mg/mL (colored in red), 0.5 mg/mL (green), and 0.3 mg/mL (blue). Calculated molecular weight of CaPLA1 from the AUC peak is 86.0 ± 2.0 kDa, which strongly suggests that CaPLA1 forms homodimers in solution, considering that the theoretical molecular weight of dimeric CaPLA1 is 90.4 kDa.

Crystal structures of AtDSEL and CaPLA1 were determined at a resolution of 1.8 Å and 2.4 Å, respectively. There are two AtDSEL molecules in the asymmetric unit (Figure 1A), and each monomer chain consists of 16 α-helices (labeled α1–α16) and 11 β-strands (labeled β1–β11) (Figure 1B). AtDSEL shows the typical structure of an α/β hydrolase; core β-strands (β1–β3 and β6–β9) are flanked by several α-helices (Figure 1B). An additional two β-strands (β4 and β5) and surrounding α-helices are also common in PLAs; however, C-terminal α-helices (α14–α16) and β-strands (β10 and β11), colored in blue, are unique features (Figure 1B). No such C-terminal domain has been found in any PLAs. Furthermore, the domain appears to be involved in dimerization; the two monomers interact with each other via the domain (Figure 1A). We used analytical ultracentrifugation (AUC) to confirm whether AtDSEL in solution is homodimerized or not. The theoretical molecular weight of dimeric AtDSEL is 95.7 kDa, and calculated molecular weight from the AUC peak is 95.3 kDa, which strongly suggests that AtDSEL forms homodimers in solution (Figure 1C). In the case of CaPLA1, there are four molecules in the asymmetric unit. Among the four molecules, two molecules make a homodimer (Figure 1D); the other two molecules make homodimers with another symmetry mate, respectively. Each monomer of CaPLA1 has an overall structure similar to that of AtDSEL; α/β hydrolase domain and C-terminal domain are almost the same as each other (Figure 1B,E). However, two additional β-strands between α5 and α7 are not found in CaPLA1 (Figure 1B,E), which is attributed to the disordered residues around the β-strands. The AUC result of CaPLA1 also suggests that CaPLA1 makes homodimers in solution, considering that the molecular weight of the theoretical dimeric CaPLA1 and calculated from the AUC peak are 90.4 kDa and 86.0 kDa, respectively (Figure 1F). Taken together, the homodimerization of AtDSEL and CaPLA1 was demonstrated using AUC, and the C-terminal domain, which is presumed to be present only in plant PLA1s, seems to be related to the dimerization; the two monomers of AtDSEL and CaPLA1 interact with each other via the domain (Figure 1A,D).

### 2.2. Conserved Motifs and Domains of AtDSEL and CaPLA1

**Figure 2:**
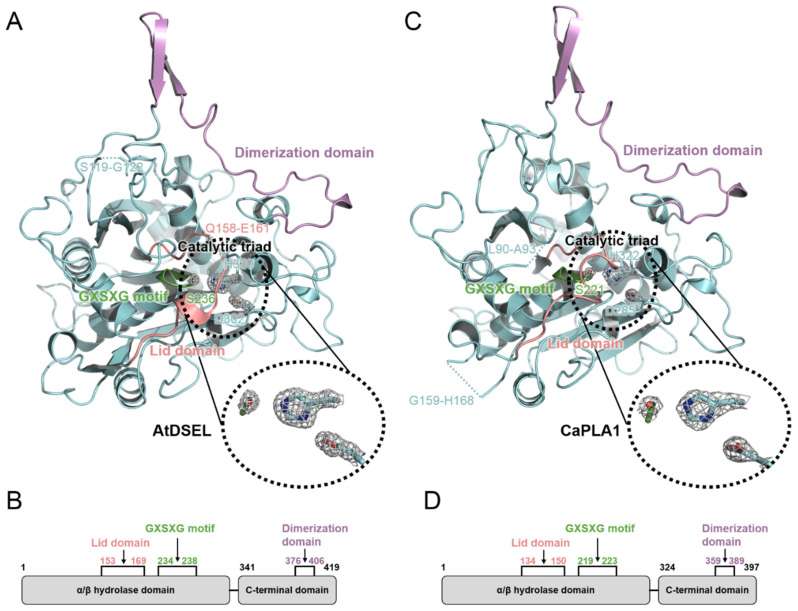
Conserved catalytic triad and domains of AtDSEL and CaPLA1. (**A**) Three residues (Ser236, Asp302, and His339) that compose the catalytic triad of AtDSEL are shown as a stick model. 2mF_O_−DF_C_ electron density map at a level of 2.0 σ is superimposed to the residues. Lid domain is colored in salmon. Pro159 and Leu160 of the lid domain were disordered, causing the disconnected lid domain in this figure. Conserved GXSXG motif is colored in green, and dimerization domain is colored in purple. (**B**) The construct map of AtDSEL. The lid domain and GXSXG motif belong to α/β hydrolase domain. C-terminal domain containing dimerization domain exists only in plant phospholipase A1 (PLA1). (**C**) Three residues (Ser221, Asp285, and His322), which compose the catalytic triad of CaPLA1, are shown as a stick model. 2mF_O_−DF_C_ electron density map at a level of 1.8 σ is superimposed to the residues. (**D**) The construct map of CaPLA1.

AtDSEL and CaPLA1 are divided into the following two domains: the α/β hydrolase domain containing the lid domain, GXSXG motif, and catalytic triad and the C-terminal domain containing the dimerization domain (Figure 2A–D). The α/β hydrolase domains of AtDSEL and CaPLA1 are composed of 340 and 323 residues, respectively, and the their C-terminal domains consist of 79 and 74 residues, respectively (Figure 2B,D). Most PLA structures reported to date have only the α/β hydrolase domain and are composed of approximately 300 residues [4,5,6]. They do not make any multimers [4,5,6]. On the other hand, mammal PLAs (human LPL and human pancreatic TGL) have another C-terminal domain in addition to the α/β hydrolase domain; the α/β hydrolase domain is composed of about 340 residues, and the other C-terminal domain consists of about 130 residues [7,8,9]. The C-terminal domain of mammals shows a typical β-barrel structure [7,8,9], which is totally different from the C-terminal domains of AtDSEL and CaPLA1. Human MGL is composed of 303 residues and has only the α/β hydrolase domain; however, the lipase makes homodimers [10]. The two monomers of human MGL makes a dimer with an up–down orientation [10]. Taken together, PLAs in fungi and venom have only the α/β hydrolase domain [4,5,6], and some PLAs in mammals and our plant PLA1s have an additional C-terminal domain as well as the α/β hydrolase domain [7,8,9] (Figure 2B,D). However, the overall structures and functions of the C-terminal domains of the PLAs in mammals and plants are completely different from each other. The C-terminal domain of AtDSEL and CaPLA1 is composed of three α-helices and one pair of β-strands (Figure 1B,E), while the C-terminal domains of PLAs in mammals are composed of 8–12 β-strands making up the β-barrel structure [7,8,9]. Moreover, the C-terminal domain of AtDSEL and CaPLA1 is involved in the homodimerization, while the β-barrel of the PLA is related to the interactions with lipids [7]. On the other hand, human MGL can make homodimers though the proteins consisting of only the α/β hydrolase domain [10]. Therefore, only the PLAs in plants have the C-terminal domain for homodimerization.

AtDSEL has two disconnected loops caused by the disordered residues; Arg120 and Glu121 between β1 and β2 and from Pro159 to Leu160 between β3 and α5 could not be assigned due to the poor electron density map of the residues (Figure 1B and Figure 2A). Pro159 and Leu160 belong to the lid domain, which determines the substrate specificities (Figure 2A,B). CaPLA1 also has two disconnected loops resulting from the disordered residues; Pro91 and Asp92 between β1 and β2 and from Asp160 to Phe167 between α6 and α7 could not be refined because of the deficient electron density map of the residues (Figure 1E and Figure 2C). The disordered residues from Asp160 to Phe167 affected the surrounding secondary structures; a pair of β-strands (β4 and β5 in the case of AtDSEL) were not found in CaPLA1 (Figure 1B,E and Figure 2A,C). It has been reported that the length of the lid domain is involved in the type of substrate that the enzyme can catalyze [3]. AtDSEL and CaPLA1 have a lid domain with the proper length for catalyzing the *sn*-1 acyl groups [3] (Figure 2B,D).

The α/β hydrolase domains of AtDSEL and CaPLA1 have a conserved GXSXG motif (Figure 2A,C). In the AtDSEL, the GXSXG motif consists of the residues from Gly234 to Gly238, while the motif is composed of the residues from Gly219 to Gly223 in the case of CaPLA1 (Figure 2B,D). The GXSXG motif is found in various lipases and esterases [22]. The central Ser of the motif functions as a nucleophile [23]. In AtDSEL, the motif exists between β6 and α9 and has the following sequence: GHSLG (Figure 1B and Figure 2A). The motif of CaPLA1 is also located in the same position and has the same sequence as in AtDSEL (Figure 2A,C). It has been reported that the two Gly residues of the GXSXG motif are also essential for the lipase activity [24]. The lipase activities of MGLs in *Arabidopsis thaliana* were completely lost when the researchers applied the following mutagenesis to the lipases: GXAXG, GXSXS, or SXSXG [24], which demonstrates that Gly to the Ser mutant as well as Ser to the Ala mutant causes severe deactivation of the lipases.

The catalytic triad of AtDSEL is Ser236, Asp302, and His339, while that of CaPLA1 is Ser221, Asp285, and His322 (Figure 2A,C). The lid domain, which is conserved in lipase proteins, plays the role of capping this active site [25] (Figure 2A,C). All human pancreatic TGL mutants of the catalytic triad (Ser, Asp, and His) showed no detectable activity or highly diminished activity [26]. Therefore, the catalytic triad could not be replaced with other residues.

### 2.3. Interactions between Homodimers of AtDSEL and CaPLA1

**Figure 3:**
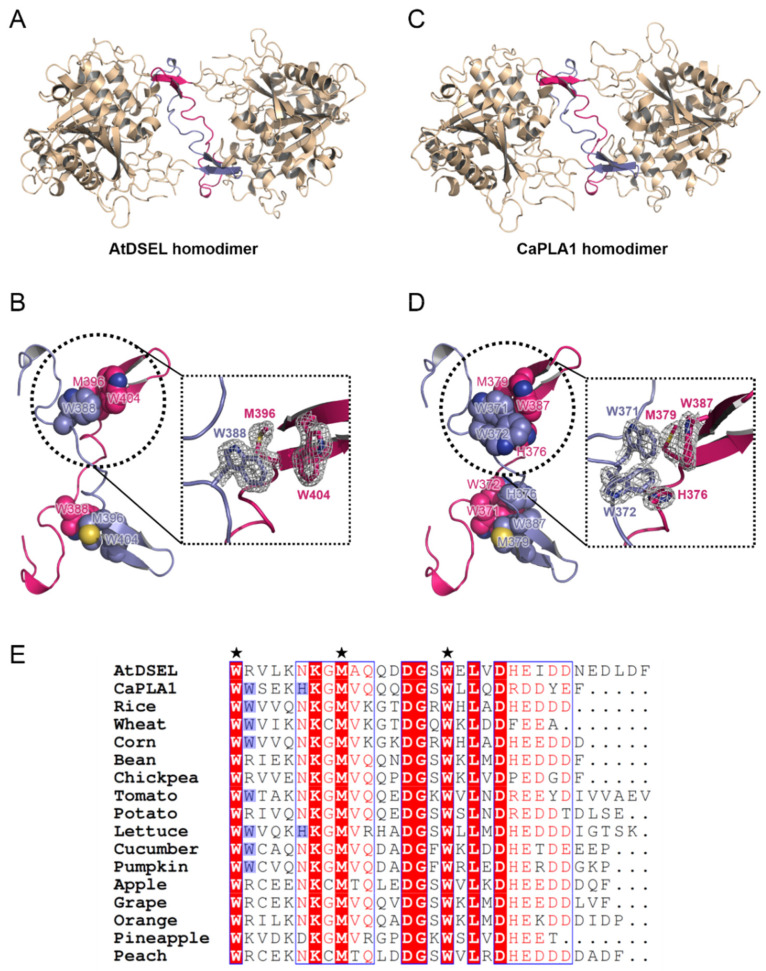
Hydrophobic interactions between homodimers of AtDSEL and CaPLA1. (**A**) AtDSEL homodimer is shown as a ribbon diagram. Each dimerization domain is colored in blue and pink, respectively. The rest is colored in wheat. (**B**) The dimerization domains of AtDSEL are shown as a ribbon diagram, and each domain is colored in blue and pink, respectively. The side-chains of Trp388, Met396, and Trp404 are shown as spheres. The side-chains of the residues are shown as a stick model in the zoomed view, and 2mF_O_−DF_C_ electron density map at a level of 2.0 σ is superimposed to the residues. (**C**) CaPLA1 homodimer is shown as a ribbon diagram. (**D**) The dimerization domains of CaPLA1 are shown as a ribbon diagram. The side-chains of Trp371, Trp372, His376, Met379, and Trp387 are shown as spheres. The side-chains of the residues are shown as a stick model in the zoomed view, and 2mF_O_−DF_C_ electron density map at a level of 1.8 σ is superimposed to the residues. (**E**) Sequence alignments of plant PLA1s, from Trp388 to C-terminal residue of AtDSEL and Trp371 to C-terminal residue of CaPLA1, were used for the alignments. In the case of other PLA1s, residues from the Trp to C-terminal were used for the alignments. The common three residues of AtDSEL and CaPLA1 involved in the hydrophobic interactions are indicated by black solid pentagrams. The additional two residues involved in the interactions of CaPLA1 are conserved in some plant PLA1s. The residues are highlighted in blue.

The dimerization domains of the AtDSEL homodimer cross each other and stretch in opposite directions (Figure 3A). Met396 and Trp404 of one monomer and Trp388 of the other monomer interact with each other (Figure 3B). The hydrophobic interactions are main driving force behind the formation of the dimerization of AtDSEL. Interactions between the monomers occur throughout the loops (Figure 3B). The length from a sulfur atom of Met396 of one monomer to a sulfur atom of the other Met396 is 23.3 Å (Figure 3B). The dimerization interface of AtDSEL is extremely long to form robust homodimers. The dimerization domain of CaPLA1 is similar to that of AtDSEL; however, more residues are involved in the dimerization (Figure 3C,D). The length from a sulfur atom of one Met379 to that of the other Met379 is 22.1 Å (Figure 3D). In CaPLA1, His376, Met379, and Trp387 of one monomer and Trp371 and Trp372 of the other monomer make hydrophobic interactions (Figure 3D). Trp372 and His376 in CaPLA1 are replaced by Arg and Asn in AtDSEL, respectively (Figure 3E). Therefore, the dimeric interface of CaPLA1 is more robust than that of AtDSEL (Figure 3B,D).

The C-terminal residues of AtDSEL, CaPLA1, and 15 PLA1s from various plants are aligned (Figure 3E). The two Trp residues and one Met of AtDSEL are conserved in the 17 PLA1s, indicating that the dimerization is commonplace in plant PLA1s (Figure 3E). Among the 17 PLA1s, eight PLA1s containing CaPLA1 have the two consecutive Trp residues, while the other PLA1s have positively charged residue instead of the second Trp (Figure 3E). His of CaPLA1 is conserved only in lettuce PLA1; the other PLA1s have Asn instead of the His, except for pineapple PLA1 (Figure 3E). The 17 PLA1s have a number of negatively charged residues in the C-terminus (Figure 3E); the reason for this has not yet been clearly elucidated. Taken together, the sequence alignments of the C-terminal regions of the plant PLA1s suggest that they would form homodimers.

### 2.4. Structural Comparison among PLAs

**Figure 4:**
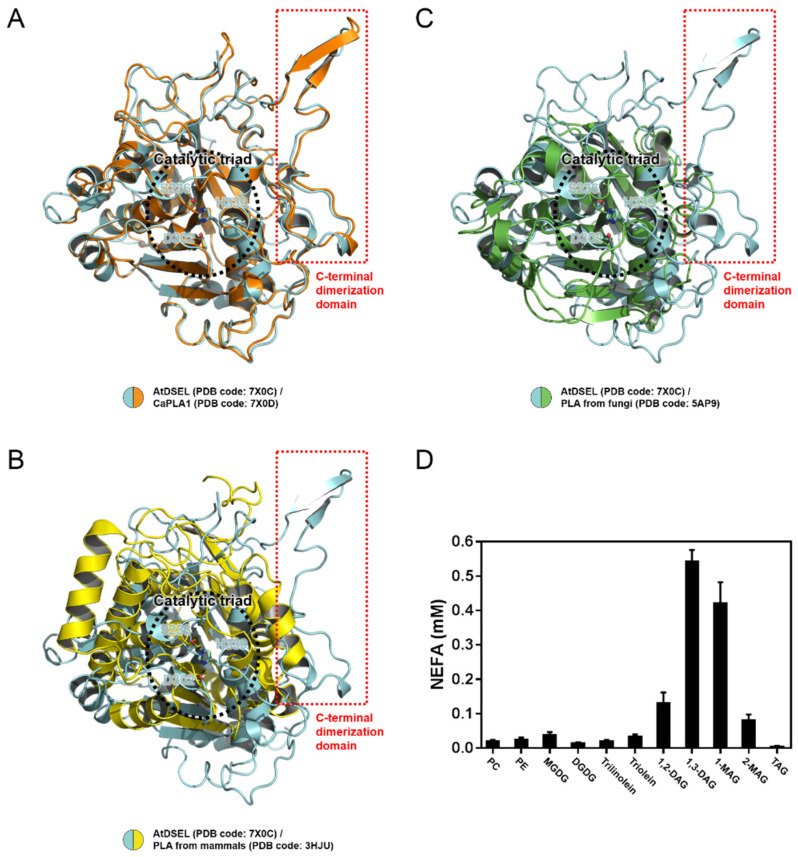
Structural comparison between AtDSEL and other PLAs, and activity assay of AtDSEL toward various lipid substrates. (**A**) Superimposition of AtDSEL and CaPLA1. AtDSEL and CaPLA1 are shown as a ribbon diagram and colored in cyan and orange, respectively. The side-chains of the catalytic triad are shown as a stick model. The additional C-terminal dimerization domains are indicated by a red dotted square. (**B**) Superimposition of AtDSEL and MGL from *Homo sapiens*. AtDSEL and MGL from *Homo sapiens* are colored in cyan and yellow, respectively. (**C**) Superimposition of AtDSEL and TGL from *Thermomyces lanuginosus*. AtDSEL and TGL from *Thermomyces lanuginosus* are colored in cyan and green, respectively. (**D**) Lipolytic activity of AtDSEL toward following substrates: phosphatidylcholine (PC), phosphatidylethanolamine (PE), monogalactosyl diacylglycerol (MGDG), digalactosyl diacylglycerol (DGDG), trilinolein, triolein, 1,2-diacylglycerol (1,2-DAG), 1,3-diacylglycerol (1,3-DAG), 1-monoacylglycerol (1-MAG), 2-monoacylglycerol (2-MAG), triacylglycerol (TAG). The released free fatty acids were measured using a nonesterified fatty acid (NEFA) colorimetric kit. Mean and standard deviation from three independent experiments are presented.

Data from multiple sequence alignments show that the C-terminal regions of AtDSEL and CaPLA1 are mostly conserved among plant PLA1s (Figure 3E); however, other PLAs with the C-terminal domain have not yet been reported. A structural comparison was performed to confirm the similarities and differences among PLAs (Figure 4A–C). AtDSEL and CaPLA1 are superimposed with an r.m.s. deviation of 1.75 Å for all Cα atoms (Figure 4A). The sequence identity between AtDSEL and CaPLA1 is 52%. The residues comprising the catalytic triad of each protein completely overlap (Figure 4A). Most of the loops as well as α-helices and β-strands are superposed (Figure 4A). In addition, the C-terminal dimerization domains of AtDSEL and CaPLA1 are very well conserved (Figure 4A). Based on the sequence alignments (Figure 3E), PLA1s of other plants are presumed to also have the conserved C-terminal dimerization domain. The substrate preference of AtDSEL toward various lipid substrates was investigated (Figure 4D). AtDSEL specifically hydrolyzed the *sn*-1 acyl chain of 1,3-diacylglycerol (1,3-DAG) and 1-monoacylglycerol (1-MAG) (Figure 4D), suggesting that AtDSEL is an *sn*-1 specific lipase, as previously known [20]. AtDSEL also showed some activity toward 1,2-diacylglycerol (1,2-DAG) and 2-monoacylglycerol (2-MAG) (Figure 4D), which are involved in the *sn*-2 acyl chain.

**Figure 5:**
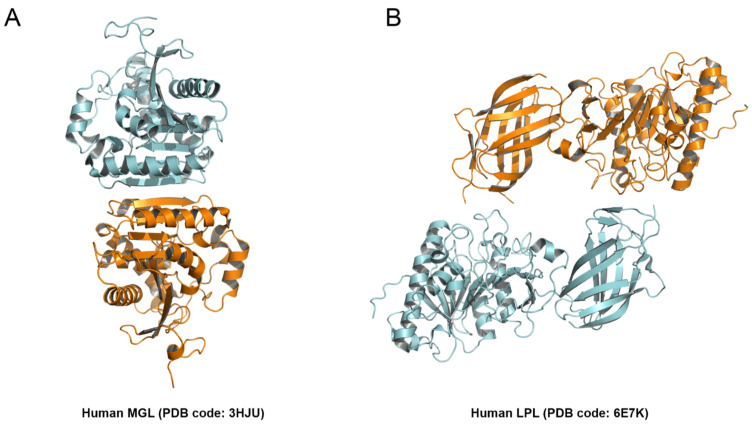
Dimerization interface of human PLAs. (**A**) Dimeric structure of human MGL. Each monomer of human MGL are colored in cyan and orange, respectively. (**B**) Dimeric structure of human LPL.

AtDSEL and human MGL are superposed with an r.m.s. deviation of 6.13 Å for all Cα atoms (Figure 4B). The sequence identity of the two proteins is very low at 13%, and even the secondary structures hardly overlap except for the core β-strands (Figure 4B). However, the residues making up the catalytic triad of human MGL are also Ser, Asp, and His, and they are well overlaid between AtDSEL and human MGL (Figure 4B). On the other hand, the C-terminal dimerization domain of AtDSEL is missing in the human MGL (Figure 4B). The human MGL forms homodimers in an up–down orientation without any additional domain, and human LPL forms homodimers in a head-to-tail orientation with the C-terminal β-barrel domain (Figure 5A,B). Therefore, the dimerization interface of the human PLAs is totally different from that of AtDSEL and CaPLA1. AtDSEL and fungi TGL are overlaid with an r.m.s. deviation of 3.93 Å for all Cα atoms (Figure 4C). Although there is a low sequence identity of 17% between the two proteins, the overall structures of them are well conserved (Figure 4C). The secondary structures of α-helices and β-strands and the catalytic triad residues of fungi TGL are highly similar to those of AtDSEL (Figure 4C). However, fungi TGL also does not have the C-terminal dimerization domain (Figure 4C).

Sequence alignments of PLA1s from various plants and structural comparison based on the structures of AtDSEL, CaPLA1, and lipases from mammals and fungi reveal that the C-terminal dimerization domain of AtDSEL and CaPLA1 is unique to plant PLAs. Thus, we propose that AtDSEL and CaPLA1 are the representative plant PLA1s, which are distinguished by the C-terminal dimerization domain for homodimerization.

## 3. Conclusions

Crystal structures of AtDSEL and CaPLA1, which are the first PLA1 structures derived from plants, reveal the unique features of the plant PLA1s. In addition to the conserved α/β hydrolase scaffold, GXSXG motif, and catalytic triad, plant PLA1s have an extended C-terminal domain composed of approximately 70 residues. In AtDSEL and CaPLA1, these C-terminal regions form a homodimer. Trp388, Met396, and Trp404 in AtDSEL and Trp371, Trp372, His376, Met379, and Trp387 in CaPLA1 play as hydrophobic anchors tethering each monomer of the proteins. A data search by BLAST indicates that the homologous C-terminal regions of AtDSEL and CaPLA1 are only found in plants. Sequence alignments of the C-terminal regions of the plant PLA1s revealed that the two Trp residues and one Met, which are the common residues between the C-terminal domains of AtDSEL and CaPLA1, are conserved. The homodimerizations of AtDSEL and CaPLA1 were supported by our AUC experiments. However, it is still unknown as to why the plant PLA1s form homodimers. The dimerization domain identified by the structures of AtDSEL and CaPLA1 can serve as the structural templates for understanding the plant phospholipase function at the molecular level. Furthermore, we found that some plant PLA1s have two Trp residues in the C-terminal region, while others have one more Trp in the C-terminal region. AtDSEL has two Trp residues and CaPLA1 has three Trp residues in the C-terminal regions, and all Trp residues in the C-terminal region play an important role as the hydrophobic anchors. Whether the difference in the number of Trp in the C-terminal region can characterize the two types of plant PLA1 dimerization domains should be further explained.

## 4. Materials and Methods

### 4.1. Cloning, Expression, and Purification

The full-length AtDSEL (Uniprot entry: O49523) and CaPLA1 (Uniprot entry: A5YW95) genes were amplified by polymerase chain reaction (PCR), respectively. The amplified cDNA fragments were subcloned into the pET21b expression vector (Novagen) with a fused 6× His tag and TEV cleavage site (ENLYFQG) at the N-terminus. The resulting plasmid was transformed into *Escherichia coli* (*E. coli*) BL21 (DE3). AtDSEL or CaPLA1 expressing cells were grown in Luria–Bertani (LB) media at 18 °C until they reached an OD_600_ of 0.7, at which time 0.5 mM isopropyl β-_D_-thiogalactopyranoside (IPTG) was added. The cells were cultured for a further 24 h at 18 °C, and stored at −80 °C after harvesting by centrifugation. Harvested cells were disrupted by sonication in a lysis buffer containing 25 mM sodium phosphate (pH 7.0), 100 mM NaCl, 5 mM β-mercaptoethanol, and protease inhibitor cocktail (Roche). The fusion protein was purified by immobilized metal affinity chromatography (IMAC) on an Ni-NTA column (Amersham Pharmacia Biotech) with the lysis buffer containing 500 mM imidazole. The purified protein was then dialyzed against the lysis buffer (25 mM sodium phosphate (pH 7.0), 100 mM NaCl, 5 mM β-mercaptoethanol). The dialyzed protein was subjected to TEV cleavage for 12 h, and then purified by IMAC on an Ni-NTA column (Amersham Pharmacia Biotech). The protein without His tag was eluted with the lysis buffer containing 20 mM imidazole. The protein containing non-native Gly at the N-terminus after the cleavage was further purified by size exclusion chromatography (SEC) using a HiLoad™ Superdex™ 200 column (GE Healthcare) with 20 mM Tris (pH 8.0), 100 mM NaCl, and 2 mM DTT and concentrated to 20 mg/mL for crystallization.

### 4.2. Crystallization, Data Collection, and Structure Determination

Crystallization screening of sitting drop was carried out with a mosquito^®^ crystallization robot (TTP Labtech) using commercial screening kits from Hampton Research (Aliso Viejo, CA) and Rigaku (Tokyo, Japan). The initial crystals of AtDSEL were observed in 100 mM sodium acetate (pH 4.6), 30% (*v*/*v*) MPD, and 20 mM calcium chloride by mixing equal volumes (each 300 nL) of protein solution (in 20 mM Tris (pH 8.0), 100 mM NaCl, and 2 mM DTT) and mother liquor. The initial crystals of CaPLA1 were observed in 100 mM HEPES (pH 7.5), 20% (*v*/*v*) PEG 8000, 10% (*v*/*v*) 2-propanol, and 200 mM ammonium sulfate by mixing equal volumes of protein solution and mother liquor. The crystals of both proteins were needle-shaped and approximately 20 μm in size. The crystals were flash-cooled to 100 K in liquid nitrogen. Diffraction data were collected using an EIGER X 16M detector on the BL-17A beamline at the Photon Factory (PF), Tsukuba, Japan. The distance from the detector to the crystal was 210 mm, and a total of 360 images were collected with oscillation widths of 1°, exposing the crystal to the beam for 1 s per image. The data were integrated and scaled using HKL2000 [27]. The diffraction data for AtDSEL and CaPLA1 were collected at a maximum resolution of 1.80 Å and 2.40 Å, respectively. The initial model of AtDSEL and CaPLA1 was determined by molecular replacement (MR) using our AtDSEL structure (PDB code: 2YIJ) with the PHENIX software suite [28]. The model was iteratively refined using COOT and PHENIX [29]. The geometry analysis was performed during the refinement using PHENIX. The final structural coordinates of AtDSEL and CaPLA1 were deposited under the following Protein Data Bank (PDB) accession codes: 7X0C and 7X0D, respectively. Data collection and refinement statistics are summarized in Table 1.

### 4.3. Analytical Ultracentrifugation (AUC) Experiment

The experiments were conducted at 20 °C using an Optima XL-I analytical ultracentrifuge (Beckman Coulter, Brea, CA, USA) with an An-50 Ti rotor. For sedimentation velocity experiments, cells with a standard Epon two-channel centerpiece and sapphire windows were used. The sample (400 μL) and the reference buffer (420 μL) were loaded into the cells. The rotor temperature was equilibrated at 20 °C in a vacuum chamber for 2 h before the start. The sedimentation velocity experiments for AtDSEL were conducted at the following three protein concentrations: 3.0 mg/mL, 1.5 mg/mL, and 0.8 mg/mL. The experiments for CaPLA1 were conducted at the following three protein concentrations: 1.1 mg/mL, 0.5 mg/mL, and 0.3 mg/mL. Changes in the concentration gradient were monitored with a Rayleigh interference optical system at 10 min intervals during sedimentation at 50 × 103 rpm. The partial specific volume of the protein, solvent density, and solvent viscosity were calculated from standard tables using the SEDNTERP software. The resulting scans were analyzed using the continuous distribution c(s) analysis module in the SEDFIT software. Sedimentation coefficient increments of 100 were used in the appropriate range for each sample. The frictional coefficient was allowed to float during fitting. The weighted average sedimentation coefficient was obtained by integrating the range of sedimentation coefficients in which the peaks were present. The values of the sedimentation coefficient were corrected to 20 °C in pure water (s20,w). The c(s) distribution was converted into c(M), which is the molar mass distribution.

### 4.4. In Vitro Lipase Assay

The in vitro lipase activity was measured as previously described with some modifications [30]. To examine phospholipase activity of AtDSEL, the recombinant fusion protein was incubated with 1-palmitoyl-2-^14^C-palmitoyl-PC (2.22 GBq/mmol; GE Healthcare, Uppsala, Sweden) in 50 mM sodium phosphate buffer (pH 5.8). Lipids were extracted and separated by thin layer chromatography (TLC) (Silica Gel 60; Merck). Radioactive products were detected using the Bio-Imaging Analyzer (BAS2500; Fuji Photo Film). To determine substrate specificity, the MBP-AtDSEL was incubated with 250 μM of various lipid substrates having *sn*-1 or *sn*-2 acyl chains, including phosphatidylcholine (PC), phosphatidylethanolamine (PE), monogalactosyl diacylglycerol (MGDG), digalactosyl diacylglycerol (DGDG), trilinolein, triolein, 1,2-diacylglycerol (1,2-DAG), 1,3-diacylglycerol (1,3-DAG), 1-monoacylglycerol (1-MAG), 2-monoacylglycerol (2-MAG), and triacylglycerol (TAG) in 200 μL of 50 mM sodium phosphate buffer (pH 7.2) for 30 min at 30 °C. The released free fatty acids were extracted and reacted using the nonesterified fatty acid (NEFA) colorimetric kit (Wako Pure Chemicals) and measured colorimetrically at 546 nm.

## Figures and Tables

**Table 1:** Summary of data collection and refinement of AtDSEL and CaPLA1.

Protein	AtDSEL			CaPLA1		
PDB Code	7X0C			7X0D		
**Data collection**						
Space group	*P12_1_1*			*P12_1_1*		
Cell dimensions						
a, b, c (Å)	73.977	95.343	79.278	102.313	59.568	147.319
α, β, γ (°)	90.000	105.144	90.000	90.000	90.139	90.000
Resolution (Å)	50–1.80 (1.86–1.80)			50–2.40 (2.44–2.40)		
R_merge_	0.086 (0.400)			0.119 (0.589)		
I/σ (I)	35.29 (4.70)			13.97 (2.79)		
Completeness (%)	99.8 (100.0)			96.1 (95.5)		
Multiplicity	7.5 (7.5)			3.5 (3.0)		
**Refinement**						
Resolution (Å)	33.29–1.80			49.11–2.40		
No. reflections	98,085			67,351		
R_work_/R_free_	0.1783/0.2117			0.2065/0.2602		
No. atoms						
Protein	6409			12,431		
Water	423			508		
B-factors (Å^2^)						
Protein	24.68			26.50		
Water	28.83			28.53		
R.m.s. deviations						
Bond lengths (Å)	0.012			0.002		
Bond angles (°)	1.127			0.545		
Ramachandran plot (%)						
Favored	98.35			97.29		
Allowed	1.65			2.45		
Disallowed	0.00			0.26		

Values in parentheses are for the highest resolution shell.

## Data Availability

The atomic coordinates for AtDSEL and CaPLA1 have been deposited in the Protein Data Bank under the accession code 7X0C and 7X0D, respectively.

## Sample Availability

Samples of the compounds are not available from the authors.
